# 8β-Acet­oxy-14α-benzo­yloxy-*N*-ethyl-3α,10β,13β,15α-tetra­hydr­oxy-1α,6α,16β-trimeth­oxy-4β-(methoxy­methyl­ene)aconitane: aconifine from *Aconitum karakolicum Rapaics*

**DOI:** 10.1107/S1600536809021436

**Published:** 2009-06-10

**Authors:** Bakhodir Tashkhodjaev, Mukhlis N. Sultankhodjaev

**Affiliations:** a S. Yunusov Institute of Chemistry of Plant Substances, Academy of Sciences of Uzbekistan, M. Ulugbek Str. 77, Tashkent 100170, Uzbekistan

## Abstract

The title compound, C_34_H_47_NO_12_, is the norditerpenoid alkaloid aconifine isolated from the leaves and tubers of *Aconitum karakolicum Rapaics*. It has a lycoctonine carbon skeleton and contains four six-membered rings and two five-membered rings; its geometry is similar to that observed in other lycoctonine-type diterpenoid alkaloids. There are two intra­molecular O—H⋯O hydrogen bonds which close five- and seven-membered pseudo-rings, respectively. In the crystal, two inter­molecular O—H⋯O hydrogen bonds cross-link the mol­ecules into double chains along the *a* axis.

## Related literature

For the isolation of aconifine, see: Sultankhodzhaev *et al.* (1973 ▶). For spectroscopic data and the chemical structure of aconifine, see: Sultankhodzhaev *et al.* (1980 ▶). For the neurocardiotoxic activity of aconifine, see: Dzhakhangirov *et al.* (1997 ▶). For the neurocardiotoxic activity of lycoctonine alkaloids, see: Dzhakhangirov *et al.* (1976 ▶). For general background to lycoctonine alkaloids and their structures, see: Joshi & Pelletier (1987 ▶).
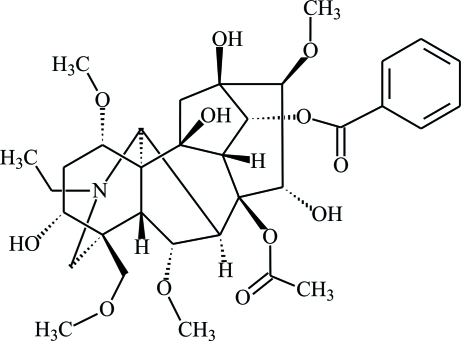


## Experimental

### 

#### Crystal data

C_34_H_47_NO_12_
*M* *_r_* = 661.73
Orthorhombic, 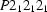

*a* = 12.0213 (3) Å
*b* = 15.4938 (6) Å
*c* = 17.1038 (4) Å
*V* = 3185.68 (16) Å^3^
*Z* = 4
Cu *K*α radiation
μ = 0.87 mm^−1^
*T* = 100 K
0.40 × 0.30 × 0.25 mm

#### Data collection

Oxford Diffraction Xcalibur Ruby diffractometer
Absorption correction: multi-scan (CrysAlisPro; Oxford Diffraction, 2009 ▶) *T*_min_ = 0.767, *T*_max_ = 0.811
11111 measured reflections
6334 independent reflections
6050 reflections with *I* > 2σ(*I*)
*R*_int_ = 0.024

#### Refinement

*R*[*F* ^2^ > 2σ(*F* ^2^)] = 0.037
*wR*(*F* ^2^) = 0.098
*S* = 1.06
6334 reflections
451 parameters
H atoms treated by a mixture of independent and constrained refinement
Δρ_max_ = 0.36 e Å^−3^
Δρ_min_ = −0.22 e Å^−3^
Absolute structure: Flack (1983 ▶), 2633 Friedel pairs
Flack parameter: 0.04 (10)

### 

Data collection: *CrysAlisPro* (Oxford Diffraction, 2009 ▶); cell refinement: *CrysAlisPro*; data reduction: *CrysAlisPro*; program(s) used to solve structure: *SHELXS97* (Sheldrick, 2008 ▶); program(s) used to refine structure: *SHELXL97* (Sheldrick, 2008 ▶); molecular graphics: *SHELXTL* (Sheldrick, 2008 ▶); software used to prepare material for publication: *SHELXTL*.

## Supplementary Material

Crystal structure: contains datablocks I, global. DOI: 10.1107/S1600536809021436/zl2215sup1.cif

Structure factors: contains datablocks I. DOI: 10.1107/S1600536809021436/zl2215Isup2.hkl

Additional supplementary materials:  crystallographic information; 3D view; checkCIF report

## Figures and Tables

**Table 1:** Hydrogen-bond geometry (Å, °)

*D*—H⋯*A*	*D*—H	H⋯*A*	*D*⋯*A*	*D*—H⋯*A*
O11—H11⋯O5	0.83 (3)	2.07 (3)	2.791 (2)	146 (3)
O8—H8⋯O12	0.83 (3)	2.11 (3)	2.598 (2)	117 (3)
O2—H2⋯O7^i^	0.87 (3)	2.21 (3)	3.066 (2)	168 (3)
O8—H8⋯O2^ii^	0.83 (3)	2.39 (3)	2.928 (2)	123 (3)

Symmetry codes: (i) 
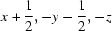
; (ii) 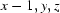.

**Fig. 1.**
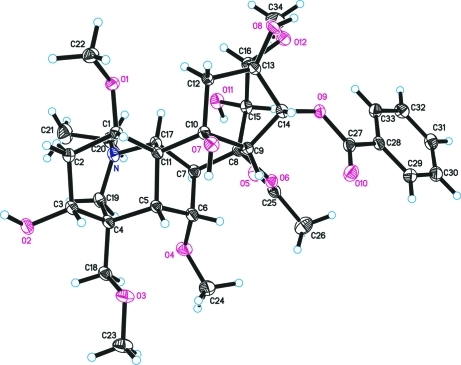
The molecular structure of aconifine, showing the atomic numbering scheme and displacement ellipsoids drawn at the 50% probability level.

**Fig. 2.**
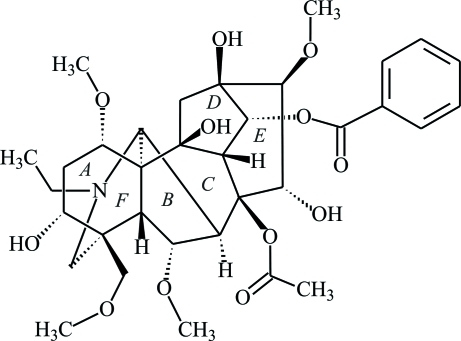
Ring assignments in Aconifine

**Fig. 3.**
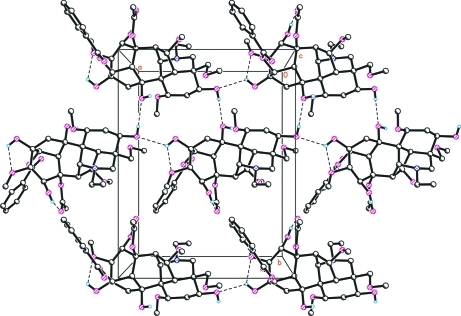
Crystal packing of aconifine, viewed down the c-axis; H-bonds shown as dashed lines. H-atoms not involved in hydrogen bonding are omitted for clarity.

## supplementary crystallographic information

### Comment

The norditerpenoid alkaloid aconifine was isolated from leaves and tubers of *Aconitum karakolicum Rapaics* (Sultankhodzhaev *et al.*,1980). It exhibits neurocardiotoxic properties (Dzhakhangirov *et al.*, 1997) similar to those of aconitine (Dzhakhangirov *et al.*, 1976). The molecular structure of the title compound is shown in Fig. 1. Aconifine has a lycoctonine carbon skeleton; its geometry is similar to that observed in other lycoctonine type diterpenoid alkaloids (Joshi *et al.*,1987).

The lycoctonine carbon skeleton, contains four six-membered rings, (*A*, *C*, *E* and *F*), and two five-membered rings (*B* and *D*) (Fig. 2). Rings *A* and *C *have more or less regular chair conformations, whereas ring *F* shows significant distortions and ring *E* adopts a sofa conformation. The five-membered rings *B *and *D* have envelope conformations.

The position and orientation of the 10 oxo substituents on the carbon lycoctonine skeleton are 1α, 3α, 4β, 6α, 8β, 10β, 13β, 14α, 15α, 16β, which confirms the earlier structure assignment based on spectral data (Sultankhodzhaev *et al.*, 1980).

In the crystal structure of the title compound there are four acidic H atoms that can participate in H-bonds. The H8 and H11 hydroxyl hydrogen atoms take part in intramolecular H-bonds which close 5 and 7-membered pseudo-cycles, respectively. Hydroxyl hydrogen atom H7 does not take a part in any H-bonding interactions. Atoms H2 and H8 participate in intermolecular O—H···O bonds which link the molecules into infinite double chains along the *a-*axis (Table 1; Fig.3). The hydrogen atom H8 forms a bifurcated H-bond to both O2 and O7.

### Experimental

The title compound was isolated from the chloroform fraction of the tubers of *Aconitum karakolicum Rapaics* by a known method (Sultankhodzhaev *et al.*, 1973). Crystals suitable for X-ray analysis were obtained by slow evaporation of a methanol solution at room temperature (m.p. 471–473 K).

### Refinement

The hydroxyl hydrogen atoms were located in a difference Fourier map and refined isotropically. The H atoms bonded to C atoms were placed geometrically (with C—H distances of 0.98 Å for CH; 0.97 Å for CH_2_; 0.96 Å for CH_3_; and 0.93 Å for C_ar_) and included in the refinement in a riding motion approximation with U_iso_=1.2U_eq_(C) [U_iso_=1.5U_eq_(C) for methyl H atoms].

### Crystal data

**Table:**

C_34_H_47_NO_12_	*D*_x_ = 1.380 Mg m^−^^3^
*M**_r_* = 661.73	Melting point: 472(2) K
Orthorhombic, *P*2_1_2_1_2_1_	Cu *K*α radiation, λ = 1.54184 Å
Hall symbol: P 2ac 2ab	Cell parameters from 11111 reflections
*a* = 12.0213 (3) Å	θ = 3.7–75.5°
*b* = 15.4938 (6) Å	µ = 0.87 mm^−^^1^
*c* = 17.1038 (4) Å	*T* = 100 K
*V* = 3185.68 (16) Å^3^	Prizmatic, colourless
*Z* = 4	0.40 × 0.30 × 0.25 mm
*F*(000) = 1416	

### Data collection

**Table:**

Oxford Diffraction Xcalibur Ruby diffractometer	6334 independent reflections
Radiation source: Enhance (Cu) X-ray Source	6050 reflections with *I* > 2σ(*I*)
graphite	*R*_int_ = 0.024
Detector resolution: 10.2576 pixels mm^-1^	θ_max_ = 75.6°, θ_min_ = 3.9°
ω scans	*h* = −15→9
Absorption correction: multi-scan (*CrysAlis PRO*; Oxford Diffraction, 2009)	*k* = −19→19
*T*_min_ = 0.767, *T*_max_ = 0.811	*l* = −21→21
11111 measured reflections	

### Refinement

**Table:**

Refinement on *F*^2^	Hydrogen site location: inferred from neighbouring sites
Least-squares matrix: full	H atoms treated by a mixture of independent and constrained refinement
*R*[*F*^2^ > 2σ(*F*^2^)] = 0.037	*w* = 1/[σ^2^(*F*_o_^2^) + (0.0771*P*)^2^] where *P* = (*F*_o_^2^ + 2*F*_c_^2^)/3
*wR*(*F*^2^) = 0.098	(Δ/σ)_max_ < 0.001
*S* = 1.06	Δρ_max_ = 0.36 e Å^−^^3^
6334 reflections	Δρ_min_ = −0.22 e Å^−^^3^
451 parameters	Extinction correction: *SHELXL97* (Sheldrick, 2008), Fc^*^=kFc[1+0.001xFc^2^λ^3^/sin(2θ)]^-1/4^
0 restraints	Extinction coefficient: 0.00043 (12)
Primary atom site location: structure-invariant direct methods	Absolute structure: Flack (1983), 2633 Friedel pairs
Secondary atom site location: difference Fourier map	Flack parameter: 0.04 (10)

### Special details

**Table:**

Geometry. All e.s.d.'s (except the e.s.d. in the dihedral angle between two l.s. planes) are estimated using the full covariance matrix. The cell e.s.d.'s are taken into account individually in the estimation of e.s.d.'s in distances, angles and torsion angles; correlations between e.s.d.'s in cell parameters are only used when they are defined by crystal symmetry. An approximate (isotropic) treatment of cell e.s.d.'s is used for estimating e.s.d.'s involving l.s. planes.
Refinement. Refinement of *F*^2^ against ALL reflections. The weighted *R*-factor *wR* and goodness of fit *S* are based on *F*^2^, conventional *R*-factors *R* are based on *F*, with *F* set to zero for negative *F*^2^. The threshold expression of *F*^2^ > σ(*F*^2^) is used only for calculating *R*-factors(gt) *etc*. and is not relevant to the choice of reflections for refinement. *R*-factors based on *F*^2^ are statistically about twice as large as those based on *F*, and *R*- factors based on ALL data will be even larger.

### Fractional atomic coordinates and isotropic or equivalent isotropic displacement parameters (Å^2^)

**Table:**

	*x*	*y*	*z*	*U*_iso_*/*U*_eq_	
O1	0.66866 (9)	−0.16533 (7)	−0.10572 (6)	0.0176 (2)	
O2	1.04961 (9)	−0.13201 (8)	−0.02826 (7)	0.0209 (2)	
O3	0.98504 (9)	−0.08240 (8)	0.16387 (7)	0.0217 (2)	
O4	0.82173 (10)	0.09161 (8)	0.13342 (7)	0.0232 (2)	
O5	0.55172 (11)	0.23615 (8)	0.08471 (7)	0.0251 (3)	
O6	0.53676 (9)	0.10653 (7)	0.14475 (6)	0.0171 (2)	
O7	0.58835 (9)	−0.18623 (7)	0.09376 (6)	0.0175 (2)	
O8	0.29051 (9)	−0.12191 (8)	−0.00458 (6)	0.0183 (2)	
O9	0.33324 (9)	0.00330 (7)	0.12982 (6)	0.0162 (2)	
O10	0.38215 (10)	−0.02601 (8)	0.25398 (6)	0.0234 (2)	
O11	0.50108 (10)	0.13929 (8)	−0.04816 (7)	0.0213 (2)	
O12	0.26888 (9)	0.04325 (8)	−0.02596 (7)	0.0210 (2)	
N	0.79454 (11)	0.02206 (9)	−0.06929 (8)	0.0169 (3)	
C1	0.73828 (12)	−0.16204 (9)	−0.03752 (8)	0.0145 (3)	
H1A	0.7261	−0.2152	−0.0076	0.017*	
C2	0.85921 (13)	−0.16088 (10)	−0.06393 (9)	0.0170 (3)	
H2A	0.8792	−0.2176	−0.0834	0.020*	
H2B	0.8673	−0.1202	−0.1066	0.020*	
C3	0.93799 (12)	−0.13616 (10)	0.00116 (9)	0.0163 (3)	
H3A	0.9347	−0.1807	0.0418	0.020*	
C4	0.90603 (12)	−0.04888 (9)	0.03803 (9)	0.0157 (3)	
C5	0.79025 (12)	−0.06011 (9)	0.07794 (8)	0.0143 (3)	
H5A	0.7935	−0.1039	0.1192	0.017*	
C6	0.74394 (13)	0.02751 (10)	0.11062 (9)	0.0170 (3)	
H6A	0.6966	0.0146	0.1558	0.020*	
C7	0.66856 (12)	0.06323 (9)	0.04426 (8)	0.0155 (3)	
H7A	0.6874	0.1233	0.0320	0.019*	
C8	0.54779 (12)	0.05540 (9)	0.07171 (8)	0.0147 (3)	
C9	0.52897 (12)	−0.03713 (10)	0.10283 (8)	0.0140 (3)	
H9A	0.5578	−0.0423	0.1562	0.017*	
C10	0.58311 (13)	−0.10691 (9)	0.04910 (8)	0.0143 (3)	
C11	0.70101 (12)	−0.08482 (10)	0.01411 (8)	0.0137 (3)	
C12	0.49078 (12)	−0.12252 (10)	−0.01330 (8)	0.0147 (3)	
H12A	0.5152	−0.1018	−0.0640	0.018*	
H12B	0.4746	−0.1837	−0.0175	0.018*	
C13	0.38627 (12)	−0.07295 (10)	0.01320 (8)	0.0156 (3)	
C14	0.40634 (12)	−0.06253 (9)	0.10058 (8)	0.0151 (3)	
H14A	0.3941	−0.1172	0.1281	0.018*	
C15	0.45606 (12)	0.08664 (10)	0.01258 (8)	0.0157 (3)	
H15A	0.4053	0.1236	0.0424	0.019*	
C16	0.38277 (13)	0.01688 (10)	−0.02702 (8)	0.0167 (3)	
H16A	0.4066	0.0104	−0.0815	0.020*	
C17	0.69192 (12)	0.00319 (9)	−0.02677 (9)	0.0149 (3)	
H17A	0.6284	0.0035	−0.0627	0.018*	
C18	0.99741 (13)	−0.02636 (10)	0.09801 (9)	0.0177 (3)	
H18A	1.0703	−0.0340	0.0747	0.021*	
H18B	0.9902	0.0333	0.1143	0.021*	
C19	0.89793 (13)	0.02424 (10)	−0.02277 (9)	0.0181 (3)	
H19A	0.9025	0.0793	0.0041	0.022*	
H19B	0.9610	0.0203	−0.0579	0.022*	
C20	0.78363 (14)	0.09877 (11)	−0.11883 (10)	0.0221 (3)	
H20A	0.7980	0.1499	−0.0878	0.026*	
H20B	0.7080	0.1024	−0.1384	0.026*	
C21	0.86362 (17)	0.09632 (14)	−0.18710 (11)	0.0345 (4)	
H21A	0.8541	0.0433	−0.2153	0.052*	
H21B	0.9386	0.1000	−0.1681	0.052*	
H21C	0.8489	0.1442	−0.2213	0.052*	
C22	0.65941 (14)	−0.24944 (10)	−0.13754 (9)	0.0204 (3)	
H22A	0.7274	−0.2644	−0.1636	0.031*	
H22B	0.5991	−0.2510	−0.1743	0.031*	
H22C	0.6454	−0.2900	−0.0963	0.031*	
C23	1.06760 (15)	−0.06738 (12)	0.22176 (10)	0.0264 (4)	
H23A	1.0660	−0.0078	0.2371	0.040*	
H23B	1.1396	−0.0811	0.2009	0.040*	
H23C	1.0530	−0.1031	0.2665	0.040*	
C24	0.84230 (16)	0.09095 (14)	0.21572 (11)	0.0320 (4)	
H24A	0.7750	0.1051	0.2431	0.048*	
H24B	0.8987	0.1327	0.2280	0.048*	
H24C	0.8670	0.0346	0.2313	0.048*	
C25	0.54687 (13)	0.19314 (10)	0.14337 (10)	0.0206 (3)	
C26	0.55057 (18)	0.22808 (12)	0.22522 (11)	0.0316 (4)	
H26A	0.6043	0.1964	0.2552	0.047*	
H26B	0.4786	0.2223	0.2489	0.047*	
H26C	0.5711	0.2879	0.2239	0.047*	
C27	0.33426 (13)	0.01855 (10)	0.20752 (9)	0.0170 (3)	
C28	0.26739 (12)	0.09676 (10)	0.22593 (9)	0.0170 (3)	
C29	0.26246 (13)	0.12388 (11)	0.30387 (9)	0.0198 (3)	
H29	0.3007	0.0937	0.3424	0.024*	
C30	0.19980 (14)	0.19638 (11)	0.32318 (9)	0.0223 (3)	
H30	0.1962	0.2147	0.3749	0.027*	
C31	0.14270 (14)	0.24149 (10)	0.26579 (10)	0.0227 (3)	
H31	0.1015	0.2902	0.2790	0.027*	
C32	0.14703 (14)	0.21384 (11)	0.18828 (10)	0.0221 (3)	
H32	0.1080	0.2437	0.1500	0.027*	
C33	0.20932 (13)	0.14203 (11)	0.16821 (9)	0.0195 (3)	
H33	0.2125	0.1239	0.1164	0.023*	
C34	0.24229 (16)	0.10857 (13)	−0.08130 (11)	0.0303 (4)	
H34A	0.2836	0.1600	−0.0694	0.045*	
H34B	0.1641	0.1208	−0.0790	0.045*	
H34C	0.2613	0.0890	−0.1329	0.045*	
H2	1.061 (2)	−0.1796 (17)	−0.0539 (15)	0.032 (6)*	
H7	0.640 (2)	−0.1782 (18)	0.1299 (17)	0.040 (7)*	
H8	0.237 (2)	−0.0881 (15)	−0.0020 (13)	0.023 (5)*	
H11	0.519 (2)	0.1832 (19)	−0.0238 (17)	0.041 (7)*	

### Atomic displacement parameters (Å^2^)

**Table:**

	*U*^11^	*U*^22^	*U*^33^	*U*^12^	*U*^13^	*U*^23^
O1	0.0172 (5)	0.0198 (5)	0.0158 (5)	0.0027 (4)	−0.0020 (4)	−0.0033 (4)
O2	0.0136 (5)	0.0231 (6)	0.0259 (5)	0.0012 (4)	0.0028 (4)	−0.0038 (5)
O3	0.0188 (5)	0.0242 (6)	0.0221 (5)	−0.0053 (5)	−0.0068 (4)	0.0021 (5)
O4	0.0197 (6)	0.0220 (5)	0.0280 (6)	−0.0039 (5)	−0.0034 (4)	−0.0074 (5)
O5	0.0278 (6)	0.0180 (5)	0.0294 (6)	−0.0022 (5)	0.0028 (5)	−0.0010 (5)
O6	0.0167 (5)	0.0162 (5)	0.0183 (5)	−0.0002 (4)	0.0021 (4)	−0.0033 (4)
O7	0.0168 (5)	0.0162 (5)	0.0196 (5)	−0.0005 (4)	−0.0001 (4)	0.0022 (4)
O8	0.0109 (5)	0.0204 (5)	0.0237 (5)	−0.0001 (5)	−0.0016 (4)	−0.0030 (4)
O9	0.0136 (5)	0.0195 (5)	0.0155 (5)	0.0026 (4)	0.0018 (4)	−0.0008 (4)
O10	0.0243 (6)	0.0280 (6)	0.0180 (5)	0.0066 (5)	0.0015 (4)	0.0020 (5)
O11	0.0229 (6)	0.0199 (5)	0.0210 (5)	0.0006 (5)	0.0027 (4)	0.0060 (5)
O12	0.0165 (5)	0.0228 (6)	0.0238 (5)	0.0051 (4)	−0.0029 (4)	0.0014 (5)
N	0.0148 (6)	0.0172 (6)	0.0188 (6)	0.0009 (5)	0.0032 (5)	0.0030 (5)
C1	0.0131 (7)	0.0155 (6)	0.0148 (6)	0.0004 (5)	−0.0011 (5)	−0.0012 (5)
C2	0.0146 (7)	0.0193 (7)	0.0171 (6)	0.0023 (6)	0.0007 (5)	−0.0032 (5)
C3	0.0115 (6)	0.0177 (7)	0.0197 (7)	0.0005 (5)	0.0006 (5)	−0.0006 (6)
C4	0.0112 (6)	0.0161 (7)	0.0199 (6)	−0.0009 (6)	0.0005 (5)	−0.0006 (5)
C5	0.0116 (6)	0.0149 (6)	0.0163 (6)	−0.0017 (5)	0.0005 (5)	−0.0002 (5)
C6	0.0134 (7)	0.0175 (7)	0.0201 (7)	−0.0005 (6)	0.0007 (5)	−0.0019 (5)
C7	0.0139 (7)	0.0149 (6)	0.0178 (6)	−0.0017 (5)	0.0012 (5)	−0.0004 (5)
C8	0.0144 (6)	0.0135 (6)	0.0163 (6)	−0.0002 (5)	−0.0002 (5)	−0.0019 (5)
C9	0.0128 (7)	0.0152 (6)	0.0140 (6)	−0.0012 (5)	0.0011 (5)	0.0001 (5)
C10	0.0136 (7)	0.0138 (6)	0.0153 (6)	−0.0004 (5)	0.0010 (5)	0.0006 (5)
C11	0.0106 (6)	0.0154 (6)	0.0152 (6)	0.0004 (5)	0.0006 (5)	−0.0001 (5)
C12	0.0113 (6)	0.0162 (7)	0.0167 (6)	0.0001 (5)	−0.0005 (5)	−0.0023 (5)
C13	0.0129 (6)	0.0170 (7)	0.0170 (6)	−0.0009 (6)	−0.0001 (5)	−0.0011 (5)
C14	0.0137 (7)	0.0149 (6)	0.0166 (6)	0.0005 (5)	0.0012 (5)	0.0015 (5)
C15	0.0153 (6)	0.0146 (6)	0.0173 (6)	0.0018 (6)	0.0015 (5)	0.0032 (5)
C16	0.0165 (7)	0.0184 (7)	0.0151 (6)	0.0032 (6)	−0.0005 (5)	−0.0008 (5)
C17	0.0124 (6)	0.0150 (6)	0.0173 (6)	0.0004 (5)	0.0010 (5)	−0.0006 (5)
C18	0.0138 (6)	0.0175 (7)	0.0217 (7)	−0.0024 (6)	−0.0002 (5)	−0.0017 (6)
C19	0.0146 (7)	0.0172 (7)	0.0225 (7)	−0.0010 (6)	0.0034 (6)	0.0022 (6)
C20	0.0224 (8)	0.0220 (8)	0.0217 (7)	0.0003 (6)	0.0033 (6)	0.0056 (6)
C21	0.0328 (10)	0.0407 (11)	0.0299 (9)	0.0001 (8)	0.0118 (8)	0.0116 (8)
C22	0.0215 (7)	0.0204 (7)	0.0194 (7)	−0.0025 (6)	−0.0008 (6)	−0.0023 (6)
C23	0.0236 (8)	0.0293 (9)	0.0263 (8)	−0.0067 (7)	−0.0086 (6)	0.0000 (7)
C24	0.0226 (8)	0.0441 (11)	0.0294 (9)	−0.0014 (8)	−0.0025 (6)	−0.0159 (8)
C25	0.0158 (7)	0.0168 (7)	0.0291 (8)	−0.0004 (6)	0.0020 (6)	−0.0034 (6)
C26	0.0426 (11)	0.0229 (8)	0.0293 (9)	0.0017 (8)	0.0006 (8)	−0.0095 (7)
C27	0.0155 (7)	0.0184 (7)	0.0171 (7)	−0.0026 (6)	0.0027 (5)	0.0007 (5)
C28	0.0131 (7)	0.0190 (7)	0.0190 (6)	−0.0017 (6)	0.0030 (5)	−0.0006 (6)
C29	0.0194 (7)	0.0213 (7)	0.0188 (7)	0.0001 (6)	0.0010 (5)	−0.0006 (6)
C30	0.0205 (8)	0.0246 (8)	0.0216 (7)	−0.0034 (7)	0.0036 (6)	−0.0048 (6)
C31	0.0217 (7)	0.0179 (7)	0.0287 (8)	0.0011 (6)	0.0053 (6)	−0.0042 (6)
C32	0.0207 (8)	0.0212 (8)	0.0245 (8)	0.0014 (6)	−0.0016 (6)	0.0024 (6)
C33	0.0192 (7)	0.0215 (7)	0.0178 (6)	−0.0017 (6)	0.0024 (5)	−0.0001 (6)
C34	0.0267 (9)	0.0285 (9)	0.0358 (9)	0.0051 (7)	−0.0108 (7)	0.0074 (8)

### Geometric parameters (Å, °)

**Table:**

O1—C22	1.4167 (18)	C10—C11	1.5762 (19)
O1—C1	1.4365 (17)	C11—C17	1.536 (2)
O2—C3	1.4346 (17)	C12—C13	1.5406 (19)
O2—H2	0.87 (3)	C12—H12A	0.9700
O3—C23	1.4211 (19)	C12—H12B	0.9700
O3—C18	1.4301 (19)	C13—C14	1.5225 (19)
O4—C6	1.4187 (19)	C13—C16	1.553 (2)
O4—C24	1.429 (2)	C14—H14A	0.9800
O5—C25	1.206 (2)	C15—C16	1.550 (2)
O6—C25	1.3476 (19)	C15—H15A	0.9800
O6—C8	1.4852 (17)	C16—H16A	0.9800
O7—C10	1.4484 (17)	C17—H17A	0.9800
O7—H7	0.89 (3)	C18—H18A	0.9700
O8—C13	1.4118 (18)	C18—H18B	0.9700
O8—H8	0.83 (3)	C19—H19A	0.9700
O9—C27	1.3500 (17)	C19—H19B	0.9700
O9—C14	1.4362 (18)	C20—C21	1.513 (2)
O10—C27	1.200 (2)	C20—H20A	0.9700
O11—C15	1.4275 (18)	C20—H20B	0.9700
O11—H11	0.83 (3)	C21—H21A	0.9600
O12—C34	1.422 (2)	C21—H21B	0.9600
O12—C16	1.4289 (18)	C21—H21C	0.9600
N—C17	1.4615 (18)	C22—H22A	0.9600
N—C20	1.466 (2)	C22—H22B	0.9600
N—C19	1.476 (2)	C22—H22C	0.9600
C1—C2	1.522 (2)	C23—H23A	0.9600
C1—C11	1.553 (2)	C23—H23B	0.9600
C1—H1A	0.9800	C23—H23C	0.9600
C2—C3	1.511 (2)	C24—H24A	0.9600
C2—H2A	0.9700	C24—H24B	0.9600
C2—H2B	0.9700	C24—H24C	0.9600
C3—C4	1.541 (2)	C25—C26	1.502 (2)
C3—H3A	0.9800	C26—H26A	0.9600
C4—C19	1.541 (2)	C26—H26B	0.9600
C4—C18	1.543 (2)	C26—H26C	0.9600
C4—C5	1.5599 (19)	C27—C28	1.488 (2)
C5—C6	1.570 (2)	C28—C33	1.398 (2)
C5—C11	1.5778 (19)	C28—C29	1.399 (2)
C5—H5A	0.9800	C29—C30	1.392 (2)
C6—C7	1.554 (2)	C29—H29	0.9300
C6—H6A	0.9800	C30—C31	1.387 (3)
C7—C8	1.531 (2)	C30—H30	0.9300
C7—C17	1.5557 (19)	C31—C32	1.394 (2)
C7—H7A	0.9800	C31—H31	0.9300
C8—C9	1.546 (2)	C32—C33	1.384 (2)
C8—C15	1.573 (2)	C32—H32	0.9300
C9—C14	1.526 (2)	C33—H33	0.9300
C9—C10	1.561 (2)	C34—H34A	0.9600
C9—H9A	0.9800	C34—H34B	0.9600
C10—C12	1.5588 (19)	C34—H34C	0.9600
			
C22—O1—C1	112.97 (11)	O11—C15—C16	107.21 (12)
C3—O2—H2	107.0 (17)	O11—C15—C8	112.21 (12)
C23—O3—C18	112.14 (12)	C16—C15—C8	117.71 (12)
C6—O4—C24	112.32 (14)	O11—C15—H15A	106.3
C25—O6—C8	120.55 (12)	C16—C15—H15A	106.3
C10—O7—H7	106.2 (18)	C8—C15—H15A	106.3
C13—O8—H8	106.3 (16)	O12—C16—C15	109.86 (12)
C27—O9—C14	117.48 (12)	O12—C16—C13	106.06 (12)
C15—O11—H11	102 (2)	C15—C16—C13	114.58 (12)
C34—O12—C16	114.23 (13)	O12—C16—H16A	108.7
C17—N—C20	111.98 (12)	C15—C16—H16A	108.7
C17—N—C19	116.56 (12)	C13—C16—H16A	108.7
C20—N—C19	111.61 (12)	N—C17—C11	110.11 (11)
O1—C1—C2	108.40 (11)	N—C17—C7	114.92 (12)
O1—C1—C11	108.72 (11)	C11—C17—C7	100.84 (11)
C2—C1—C11	115.78 (12)	N—C17—H17A	110.2
O1—C1—H1A	107.9	C11—C17—H17A	110.2
C2—C1—H1A	107.9	C7—C17—H17A	110.2
C11—C1—H1A	107.9	O3—C18—C4	108.21 (12)
C3—C2—C1	112.51 (12)	O3—C18—H18A	110.1
C3—C2—H2A	109.1	C4—C18—H18A	110.1
C1—C2—H2A	109.1	O3—C18—H18B	110.1
C3—C2—H2B	109.1	C4—C18—H18B	110.1
C1—C2—H2B	109.1	H18A—C18—H18B	108.4
H2A—C2—H2B	107.8	N—C19—C4	113.58 (12)
O2—C3—C2	109.83 (12)	N—C19—H19A	108.8
O2—C3—C4	109.72 (12)	C4—C19—H19A	108.8
C2—C3—C4	111.57 (12)	N—C19—H19B	108.8
O2—C3—H3A	108.6	C4—C19—H19B	108.8
C2—C3—H3A	108.6	H19A—C19—H19B	107.7
C4—C3—H3A	108.6	N—C20—C21	111.65 (15)
C3—C4—C19	112.64 (12)	N—C20—H20A	109.3
C3—C4—C18	107.04 (12)	C21—C20—H20A	109.3
C19—C4—C18	109.11 (12)	N—C20—H20B	109.3
C3—C4—C5	107.68 (11)	C21—C20—H20B	109.3
C19—C4—C5	108.72 (12)	H20A—C20—H20B	108.0
C18—C4—C5	111.68 (12)	C20—C21—H21A	109.5
C4—C5—C6	112.07 (12)	C20—C21—H21B	109.5
C4—C5—C11	109.32 (11)	H21A—C21—H21B	109.5
C6—C5—C11	102.42 (11)	C20—C21—H21C	109.5
C4—C5—H5A	110.9	H21A—C21—H21C	109.5
C6—C5—H5A	110.9	H21B—C21—H21C	109.5
C11—C5—H5A	110.9	O1—C22—H22A	109.5
O4—C6—C7	109.63 (12)	O1—C22—H22B	109.5
O4—C6—C5	117.99 (12)	H22A—C22—H22B	109.5
C7—C6—C5	104.75 (12)	O1—C22—H22C	109.5
O4—C6—H6A	108.0	H22A—C22—H22C	109.5
C7—C6—H6A	108.0	H22B—C22—H22C	109.5
C5—C6—H6A	108.0	O3—C23—H23A	109.5
C8—C7—C6	107.51 (11)	O3—C23—H23B	109.5
C8—C7—C17	111.31 (12)	H23A—C23—H23B	109.5
C6—C7—C17	104.61 (12)	O3—C23—H23C	109.5
C8—C7—H7A	111.1	H23A—C23—H23C	109.5
C6—C7—H7A	111.1	H23B—C23—H23C	109.5
C17—C7—H7A	111.1	O4—C24—H24A	109.5
O6—C8—C7	107.49 (11)	O4—C24—H24B	109.5
O6—C8—C9	101.06 (11)	H24A—C24—H24B	109.5
C7—C8—C9	108.54 (12)	O4—C24—H24C	109.5
O6—C8—C15	108.32 (11)	H24A—C24—H24C	109.5
C7—C8—C15	116.32 (12)	H24B—C24—H24C	109.5
C9—C8—C15	113.84 (12)	O5—C25—O6	124.70 (15)
C14—C9—C8	111.83 (12)	O5—C25—C26	125.10 (15)
C14—C9—C10	102.08 (11)	O6—C25—C26	110.20 (14)
C8—C9—C10	112.24 (11)	C25—C26—H26A	109.5
C14—C9—H9A	110.1	C25—C26—H26B	109.5
C8—C9—H9A	110.1	H26A—C26—H26B	109.5
C10—C9—H9A	110.1	C25—C26—H26C	109.5
O7—C10—C12	105.10 (11)	H26A—C26—H26C	109.5
O7—C10—C9	107.18 (11)	H26B—C26—H26C	109.5
C12—C10—C9	102.33 (11)	O10—C27—O9	123.76 (14)
O7—C10—C11	110.21 (12)	O10—C27—C28	126.00 (14)
C12—C10—C11	114.45 (11)	O9—C27—C28	110.24 (13)
C9—C10—C11	116.62 (12)	C33—C28—C29	120.07 (14)
C17—C11—C1	116.46 (12)	C33—C28—C27	121.94 (14)
C17—C11—C10	107.55 (11)	C29—C28—C27	117.98 (14)
C1—C11—C10	107.94 (12)	C30—C29—C28	119.43 (15)
C17—C11—C5	98.50 (11)	C30—C29—H29	120.3
C1—C11—C5	112.60 (12)	C28—C29—H29	120.3
C10—C11—C5	113.66 (11)	C31—C30—C29	120.43 (15)
C13—C12—C10	107.57 (11)	C31—C30—H30	119.8
C13—C12—H12A	110.2	C29—C30—H30	119.8
C10—C12—H12A	110.2	C30—C31—C32	119.99 (15)
C13—C12—H12B	110.2	C30—C31—H31	120.0
C10—C12—H12B	110.2	C32—C31—H31	120.0
H12A—C12—H12B	108.5	C33—C32—C31	120.20 (15)
O8—C13—C14	113.43 (12)	C33—C32—H32	119.9
O8—C13—C12	109.50 (12)	C31—C32—H32	119.9
C14—C13—C12	102.26 (11)	C32—C33—C28	119.88 (14)
O8—C13—C16	111.35 (12)	C32—C33—H33	120.1
C14—C13—C16	110.12 (12)	C28—C33—H33	120.1
C12—C13—C16	109.79 (12)	O12—C34—H34A	109.5
O9—C14—C13	108.67 (11)	O12—C34—H34B	109.5
O9—C14—C9	113.52 (12)	H34A—C34—H34B	109.5
C13—C14—C9	101.84 (11)	O12—C34—H34C	109.5
O9—C14—H14A	110.8	H34A—C34—H34C	109.5
C13—C14—H14A	110.8	H34B—C34—H34C	109.5
C9—C14—H14A	110.8		

### Hydrogen-bond geometry (Å, °)

**Table:**

*D*—H···*A*	*D*—H	H···*A*	*D*···*A*	*D*—H···*A*
O11—H11···O5	0.83 (3)	2.07 (3)	2.791 (2)	146 (3)
O8—H8···O12	0.83 (3)	2.11 (3)	2.598 (2)	117 (3)
O2—H2···O7^i^	0.87 (3)	2.21 (3)	3.066 (2)	168 (3)
O8—H8···O2^ii^	0.83 (3)	2.39 (3)	2.928 (2)	123 (3)

Symmetry codes:  (i) *x*+1/2, −*y*−1/2, −*z*; (ii) *x*−1, *y*, *z*.
